# Distinct gene-expression profiles characterize mammary tumors developed in transgenic mice expressing constitutively active and C-terminally truncated variants of STAT5

**DOI:** 10.1186/1471-2164-10-231

**Published:** 2009-05-18

**Authors:** Tali Eilon, Itamar Barash

**Affiliations:** 1 Institute of Animal Science, ARO, The Volcani Center, Bet-Dagan, Israel

## Abstract

**Background:**

Stat5 is a latent transcription factor that regulates essential growth and survival functions in normal cells. Constitutive activity of Stat5 and the involvement of its C-terminally truncated variant have been implicated in blood cell malignancies and mammary or breast cancer. To distinguish the individual contributions of the Stat5 variants to mammary tumorigenesis, global gene-expression profiling was performed on transgenic STAT5-induced tumors.

**Results:**

We identified 364 genes exhibiting differential expression in mammary tumors developed in transgenic mice expressing constitutively active STAT5 (STAT5ca) vs. its C-terminally truncated variant (STAT5Δ750). These genes mediate established Stat5 effects on cellular processes such as proliferation and cell death, as well as yet-unrelated homeostatic features, e.g. carbohydrate metabolism. A set of 14 genes linked STAT5Δ750 expression to the poorly differentiated carcinoma phenotype and STAT5ca to the highly differentiated papillary adenocarcinoma.

Specifically affected genes exhibited differential expression in an individual tumor set vs. its counterpart and the intact mammary gland: 50 genes were specifically affected by STAT5ca, and 94% of these were downregulated, the latter involved in suppression of tumor suppressors and proliferation antagonistics. This substantial downregulation distinguishes the STAT5ca-induced tumorigenic consequences from the relatively equal effect of the STAT5Δ750 on gene expression, which included significant elevation in the expression of oncogenes and growth mediators.

STAT5Δ750 mRNA expression was below detection levels in the tumors and the amount of STAT5ca transcript was not correlated with the expression of its specifically affected genes. Interestingly, we identified several groups of three to eight genes affected by a particular STAT5 variant with significant correlated expression at distinct locations in the clustergram.

**Conclusion:**

The different gene-expression profiles in mammary tumors caused by the STAT5Δ750 and STAT5ca variants, corroborated by the absence of a direct link to transgenic STAT5 expression, imply distinct metabolic consequences for their oncogenic role which probably initiate early in tumor development. Tumorigenesis may involve induction of growth factor and oncogenes by STAT5Δ750 or suppression of tumor suppressors and growth antagonists by STAT5ca. The list of genes specifically affected by the STAT5 variants may provide a basis for the development of a marker set for their distinct oncogenic role.

## Background

Stat5 is a latent transcription factor that is activated upon binding of specific cytokines to their cognate membranal receptors. Activation of Stat5 involves phosphorylation of specific tyrosine residues by Janus kinase 2 (Jak2), and the translocation of dimerized Stat5 molecules into the nucleus to bind target sites (TTCC(A>T)GGAA) in individual gene promoters. Consequently, induction of a wide variety of signaling events ensues, observed mainly in the hematopoietic system and mammary gland. Stat5's transactivation domain (TAD) has been mapped to amino acids 750 to 772 in the C-terminal domain of the molecule [1]. This domain interacts with co-factors and is essential for transcription activation [2]. The TAD encompasses the main diversity between STAT5a, which is prevalent in the mammary gland, and Stat5b which is more abundant in the liver. Several naturally occurring C-terminally truncated variants of Stat5 have been identified. These variants are generated by alternative splicing or proteolytic cleavage, most commonly by nuclear serine proteases [3-5].

In the blood, naive T cells in the peripheral blood mononuclear cell fraction exclusively express the C-terminally truncated Stat5 [6]. Upon activation by mitogenic stimuli, the truncated Stat5a and Stat5b are replaced by the full-length Stat5, implying that the truncated proteins have distinct functions. Naturally truncated forms of Stat5 have been implicated in blood-cell cancer: 94% of patients with relapsed leukemia expressed this Stat5 variant, suggesting that it controls progression of the disease [7]. Constitutive activation of Stat5 has also been linked to a variety of blood-derived malignancies such as BCR-ABL-induced chronic myeloid leukemia (CML [8]), acute myeloid leukemia (AML) and acute lymphoid leukemia (ALL [9]).

In the mammary gland, Stat5's expression and activity are induced during pregnancy and lactation and decrease upon involution. During these stages, Stat5 controls epithelial cell proliferation, final differentiation, lactogenesis, cell survival and tissue remodeling. These cellular processes play a major role in the structural and functional adaptation of the gland to the specific stages of the female reproductive cycle (reviewed in [10]). Overexpression of STAT5 in transgenic mice induced proliferation of mammary epithelial cells during pregnancy, increased β-casein synthesis upon lactation, and delayed involution [11]. In contrast, the C-terminally truncated variant was unable to induce β-casein/luciferase activity upon prolactin stimulation in vitro. Its expression in the mammary glands of transgenic animals resulted in reduced rates of cell proliferation at pregnancy and increased apoptosis during involution. Morphological signs of milk secretion upon parturition were delayed [12].

Stat5 has also been associated with breast cancer. Stat5a nuclear localization was observed in 76% of breast cancer specimens and a positive correlation was established between its nuclear localization and the level of histological differentiation of the tumors [13]. Inactivation of Stat5a in transgenic mice expressing transforming growth factor (TGF) α or the SV40 T antigen delayed hyperplasia [14] and mammary cancer progression. More recently, a direct effect for Stat5 on mammary tumorigenesis was established [15,16]. Overexpression and forced activation of STAT5 caused parity-dependent development, most frequently of differentiated tumors, in transgenic post-estropausal female mice. Surprisingly, comparable rates of tumors (~8%) with similar latency periods were monitored in mice expressing the C-terminally truncated STAT5 protein, though the amount of poorly differentiated tumors in these mice was higher.

Both the constitutively active and C-terminally truncated variants of Stat5 are potent oncogenes. In this study, we sought to determine the distinctness of their effects and the exclusive contribution of each variant to the oncogenic profiles of gene expression.

Profiling global gene expression in breast cancer has improved our understanding of the clinical diversity of this disease, allowing a better classification of its subtypes and definition of their response to drug treatment. Attempts to predict survival rates have also been reported [17-20]. Transgenic mouse models have been used to study the signatures of specific genes involved in initiation and maintenance of the disease. Genetic analysis of these mouse models could be highly relevant to human cancer [21-23]. The data also suggest that genes involved in the same pathway generate tumors with similar expression profiles, which are distinct from the profiles of tumors arising from other transgenic pathways [24].

Gene-expression profiles were compared in tumors caused by the constitutively active STAT5 (STAT5ca) and its C-terminally truncated variant (STAT5Δ750). A set of 364 differentially expressed genes was identified. Analysis and classification of these genes suggest that defined differences, possibly triggered at early stages of tumor development, characterize these Stat5 variants.

## Methods

### Mouse mammary-tumor samples

Mammary tumors were derived from transgenic mice carrying one of two Stat5 variants on a FVB/N background: (i) constitutively activated STAT5, termed STAT5ca, comprising sequences from three genes: amino acids 1–750 from ovine Stat5, which is homologous to mouse Stat5a, 677–847 from human Stat6, and 757–1129 from mouse Jak2 and (ii) a deleted construct, STAT5Δ750, prepared by introducing a stop codon at the respective site of the native Stat5 DNA sequence, thus eliminating the expression of its TAD. These constructs were inserted into the β-lactoglobulin (BLG) multiple-cloning site for mammary-gland-specific expression [11]. Upon identification, tumors were excised and snap-frozen for RNA isolation and validation studies. The pathological analysis of the tumors was performed by Dr. Robert Cardiff (University of California, Davis) as previously described [15]. All animals used in this study were treated humanely. Study protocols were in compliance with the regulations of the Israeli Ministry of Health and local institutional policies (approval no. IL- 39-03).

### RNA extraction and microarray hybridization

The protocols for RNA extraction from tumors and mammary glands and microarray hybridization were as previously described [23]. Briefly, RNA was extracted from individual tumors or mammary glands with TRIZOL and reverse-transcribed. Equal amounts of complementary (c) RNA from each tumor were hybridized to an Affymetrix GeneChip^® ^Mouse Genome 430A 2.0 array (Affymetrix, Santa Clara, CA), which includes approximately 14,000 annotated genes from the mouse genome. Hybridization and signal quantitation were performed according to Affymetrix's protocol by the Biological Services of the Weizmann Institute of Science (Rehovot, Israel). Total RNA (15 μg) was reversed-transcribed using a T7-oligo(dT) promoter-primer in the first-strand DNA-synthesis reaction. Following RNase H-mediated second-strand cDNA synthesis, the double-stranded cDNA was purified and used as a template for the subsequent in-vitro transcription reaction. This reaction was carried out in the presence of T7-RNA polymerase and a biotinylated nucleotide analogue/ribonucleotide mix for cRNA amplification and biotin labeling. The biotinylated cRNA targets were then cleaned up, fragmented, and hybridized to the GeneChip expression array. The chip was reacted with streptavidin-phycoerythrin and then with biotinylated anti-streptavidin antibody (Vector Laboratories, Burlingame, CA). Arrays were scanned by GeneArray scanner G2500A (Hewlett Packard, Palo Alto, CA), visually inspected for hybridization imperfections and analyzed using Affymetrix Microarray Suite software version 5.0 by scaling to an average intensity of 250. The raw data have been deposited in the public repository "Gene Expression Omnibus" (GEO), accession no. GSE15119.

### Statistical, hierarchical clustering and functional annotation analyses

The data were analyzed with GeneSpring (Silicon Genetics, Redwood City, CA) using the MAS5 algorithm [25]. Gene-expression data were normalized "per chip" and "per gene". For "per chip" normalization, all expression data on a chip were normalized to the 50^th ^percentile of the measurements taken from all values on that chip. For "per gene" normalization, each gene's measurement in the selected samples was divided by the median of the gene's measurements in the respective control group, according to the type of comparison being made. When profiles were compared between tumors induced by each of the two transgenic Stat5 variants, the expression of a given gene was normalized to the median of the expression level in the wild-type mammary gland samples. This allowed relating the level of expression of a given gene to that in the mammary gland [23]. When the analysis also included values obtained from the mammary glands, the expression of a given gene was normalized to the median of the expression levels of all genes from all samples. The normalized data were log-transformed and the differences in gene expression (based on the individual values obtained from each tumor) were calculated using one-way statistical analysis of variance (ANOVA). The statistical analysis, which discriminates between the effects of transgenic STAT5 variants, was cross-validated by the K-nearest-neighbor algorithm using the leave-one-out methodology [26]. The differences in gene expression between the tumors and the mammary gland were determined by post-hoc Student-Newman- Keuls analysis. Note that the different expression patterns of selected genes in the array had been previously confirmed by semi-quantitative PCR analyses of selected genes, and hybridization to the respective probes [23].

Genes exhibiting a significant (*P *≤ 0.05) twofold difference in expression between transgenic variants were categorized into "biological functions and/or diseases" using Ingenuity Pathways Analysis (IPA) software (Ingenuity Systems, Mountain View, CA). The probability of each term being identified by random chance was calculated using Fisher's exact test . Unsupervised hierarchical clustering (GeneSpring) organized these genes according to their similarity or dissimilarity in expression profiles, placing the cases with similar expression profiles together as neighboring rows in the clustergram.

Multivariate correlations were determined between the expressions of transgenic STAT5 and specifically affected genes, and among the expressions of the affected genes in the individual tumors using JMP statistical software (Version 7.0, SAS Institute, Inc., Cary, NC).

### Real-time PCR

Quantitative real-time PCR analyses were performed in an ABI Prism 7700 (Applied Biosystems, Foster City, CA) in a 20-μl reaction volume containing 4 μl cDNA (diluted 1:100), 10 μl SYBR Green PCR Master Mix (Applied Biosystems) and 10 μM primers. The thermal-cycle conditions consisted of 2 min at 50°C, 2 min at 95°C, and 40 cycles of 15 s at 95°C and 1 min at 60°C. The primers were designed so that the PCR would yield a single product without any primer dimerization, and the product was verified using a dissociation protocol [see Additional file 1, data sheet A]. The primers were designed across exon-exon junctions to ensure that there was no genomic DNA contamination in the cDNA samples. The amplification curves for the selected genes (70–100 bp) were parallel.

## Results

### Establishment of distinct expression profiles for the effects of STAT5ca and STAT5Δ750 in mammary tumors

**Figure 1:**
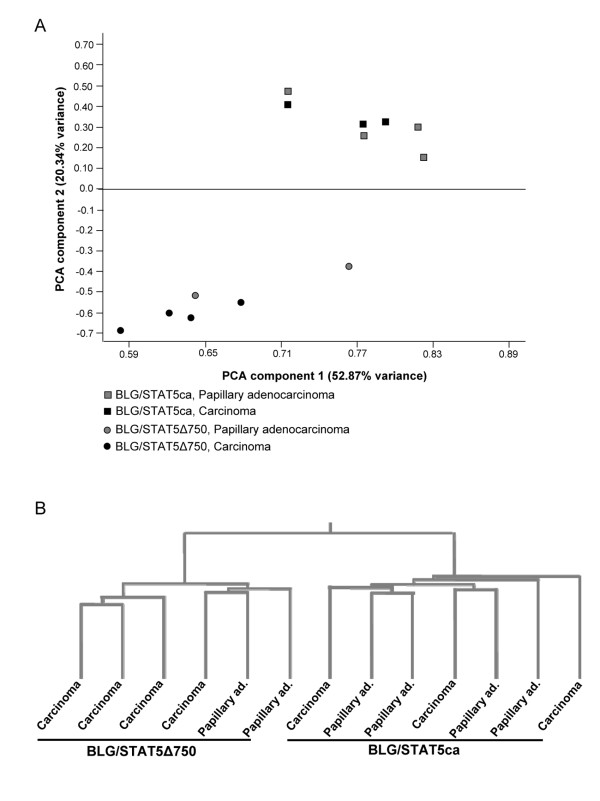
**Principal component analysis (PCA) and unsupervised hierarchical clustering into distinct tumor genotypes**. Mammary carcinomas and papillary adenocarcinomas were developed in transgenic mice expressing constitutively activated STAT5 (STAT5ca) or truncated STAT5 (STAT5Δ750). PCA (A) and unsupervised hierarchical clustering (standard correlation, B) were performed on genes that were expressed at significantly (*P *< 0.05; twofold) different levels in tumors induced by the two STAT5 variants. Both analyses confirmed their distinction based on the transgenic STAT5 variant carried by the host female.

RNA was independently extracted from six tumors developed in mice carrying the BLG/STAT5Δ750 transgene and from seven tumors originating in mice carrying BLG/STAT5ca. Tumor phenotypes were either poorly differentiated carcinoma or papillary adenocarcinoma (Fig. 1 and described in [15]). Following microarry hybridization, we identified a set of 381 features (364 genes) exhibiting a significant (*P < 0.05*) twofold difference in expression level between tumors originated from mice carrying the two transgenic STAT5 variants [see Additional file 1, data sheet B]. Their grouping according to type of transgenic STAT5 variant was confirmed by principal component analysis (PCA, [27]) and unsupervised hierarchical clustering (Fig. 1).

IPA software was used to annotate the differentially expressed genes into processes involved in cellular metabolism and cancer (Table 1). In both categories, the highest number of differentially expressed genes in the STAT5ca- vs. STAT5Δ750-induced tumors was associated with two opposing processes: cell proliferation and cell death [see Additional file 1, data sheet C]. The involvement of some of the genes affecting cancer cell death–Adm, Cflar, Cyr61, Ddit4, Itgb1, Mapk1, Mapk8, Tgfβ2, Tpm1, Vegfα and Wasf1, was demonstrated in breast cancer cell lines [see Additional file 1, data sheet C]. The activities of Itgb1 (coding for integrin β1), TGFβ2 and Vegfα are mediated, at least in part, by Cyr61 (CCN1, [28-30])–a secreted matrix protein involved in the clinical progression of breast cancer to an invasive phenotype [31].

Cyr61 can be also found among the differentially expressed genes regulating the formation of cellular protrusions and filopodia–cellular extensions needed for cellular interactions and movement [32]. Most of the listed genes affecting this process, including the laminin α5 (Lama5) [33] and the VEGF receptor PVR [34], were expressed at higher levels in the STAT5Δ750-induced tumors. This suggests higher involvement of this variant in cell movement and tumor extension. The GDP/GTP exchange protein Fabrin 4 (FDG4, [34] and the ganglioside GD3-synthase (ST8SIA)1 which marks ER-negative breast cancer tumors [35] were the only genes in this context that were expressed at a higher level in the STAT5ca-induced tumors.

**Table 1:** Cellular metabolism and cancer processes affected by genes that are differentially expressed in tumors caused by the different transgenic STAT5 variants.

**Category**	**Process**	**Process Annotation**	**Significance**	**No. of Molecules**
Carbohydrate Metabolism	formation	formation of inositol phosphate	1.53^-2^	3
	production	production of carbohydrate	8.32^-3^	7
	transport	transport of carbohydrate	1.66^-5^	11
	utilization	utilization of carbohydrate	1.04^-2^	3

Cardiovascular System Development and Function	angiogenesis	angiogenesis of cells	1.69^-3^	5
	cardiovascular process	cardiovascular process of cornea	6.15^-4^	6

Cell cycle	G1 phase	G1 phase of eukaryotic cells	1.88^-2^	11
	G2 phase	G2 phase of tumor cell lines	1.59^-2^	5
	interphase	interphase of eukaryotic cells	1.36^-2^	17
	length	length of telomeres	1.72^-2^	3
	mitogenesis	mitogenesis	3.30^-3^	10

Cell death	cell death	cell death	1.43^-3^	74
	cell death	cell death of cell lines	5.03^-4^	49
	cell death	cell death of tumor cell lines	2.11^-4^	41

Cellular Assembly and Organization	formation	formation of cellular protrusions	1.18^-2^	7
		formation of filopodia	7.48^-4^	8
		formation of plasma membrane projections	1.77^-3^	13
	growth	growth of plasma membrane projections	1.21^-2^	13

Cellular Growth and Proliferation	formation	formation of eukaryotic cells	2.93^-3^	13
	growth	growth of cell lines	4.41^-4^	36
	proliferation	proliferation of eukaryotic cells	1.58^-6^	65

Cellular Movement	cell movement	cell movement of eukaryotic cells	1.25^-3^	45
		cell movement of tumor cell lines	1.23^-4^	22
	homing	homing of eukaryotic cells	5.69^-3^	17
	migration	migration of tumor cell lines	2.23^-4^	17

DNA Replication, Recombination and Repair	synthesis	synthesis of DNA	3.78^-3^	17

Hematological System Development and Function	hematological process	hematological process	7.64^-3^	24

Nervous System Development and Function	growth	growth of neurites	1.02^-2^	13

Tissue Morphology	contraction	contraction of tissue	6.01^-4^	12

Cancer	benign tumor	benign tumor	1.55^-2^	10
	carcinoma in situ	carcinoma in situ	1.77^-2^	6
	apoptosis	apoptosis of tumor cell lines	2.33^-4^	37
	cell death	cell death of tumor cell lines	2.11^-4^	41
		cell death of breast cancer cell lines	1.12^-2^	11
	cell movement	cell movement of tumor cell lines	1.23^-4^	22
	growth	growth of tumor	2.03^-3^	10
		growth of tumor cell lines	1.01^-2^	25
	metastasis	migration of tumor cell lines	2.23^-4^	17
	proliferation	proliferation of tumor cell lines	1.29^-4^	28
	transformation	transformation of cells	1.31^-2^	19

The differential expression of genes mediating carbohydrate metabolism indicates involvement of the STAT5 variants in cellular homeostasis as well. Most of the genes involved in the transport and utilization of carbohydrates were more highly expressed in tumors caused by the BLG/STAT5Δ750 transgene, for example, Aqp7 and Aqp9 which code for proteins operating as glycerol channels [36], or glucokinase (Gck) which encodes a protein that catalyzes the conversion of glucose to glucose-6-phosphate, thus maintaining glucose homeostasis [32].

### Hierarchical clustering of genes affected by transgenic STAT5 variants

**Figure 2:**
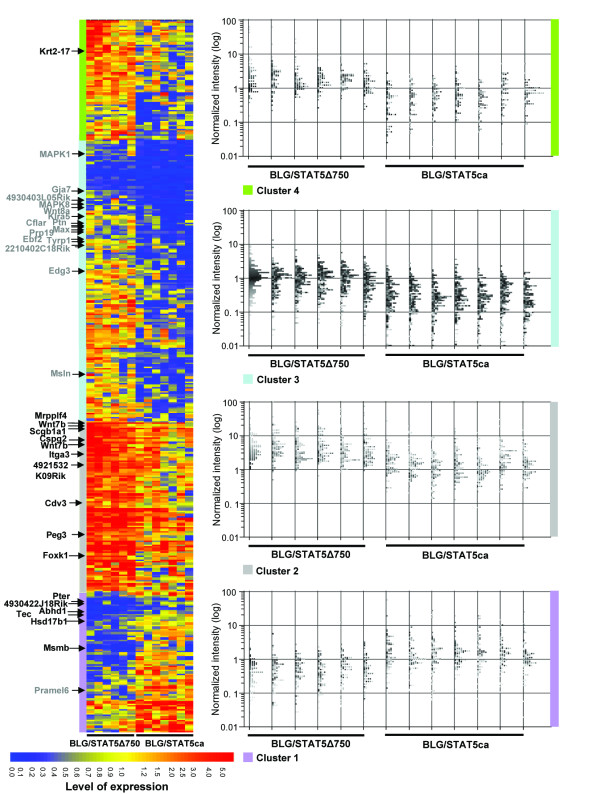
**Unsupervised hierarchical clustering of genes that are differentially expressed in tumors induced by STAT5ca and STAT5Δ750 transgenes**. Genes with statistically significant (*P *< 0.05), over twofold differences in expression levels between the two sets of tumors were clustered. In each cluster, cellular processes were annotated. The normalized intensity values indicate relative expression levels compared to the median gene expression in the intact mammary gland ("1"). Arrows mark genes that are specifically affected by STAT5ca (gray) or STAT5Δ750 (black) and listed in Table 2.

Unsupervised hierarchical clustering assembled the genes that were differentially expressed between the two sets of STAT5-affected tumors into four clusters (Fig. 2 and [see Additional file 1, data sheet D]). Cluster 1 contains genes that were more highly expressed in the STAT5ca-induced tumors than in their STAT5Δ750 counterparts, resulting from downregulation of the STAT5Δ750-induced genes compared to their expression in the mammary gland (represented by the value "1"). In contrast, genes allocated to clusters 2, 3 and 4 were more highly expressed in the STAT5Δ750-induced tumors. In cluster 2, the STAT5Δ750 affected genes were, in general, also more highly expressed than in the wild-type gland. In cluster 3, the STAT5Δ750-affected genes maintained mammary gland levels of expression while their STAT5ca counterparts were expressed at a lower level. Finally, in cluster 4, differential expression relative to the mammary gland was noted: higher expression of genes induced by the BLG/STAT5Δ750 transgene, lower expression of genes affected by its BLG/STAT5ca counterpart.

Annotation of the 76 genes in cluster 1 assembled them into non-malignant cellular processes, including amino acid modification, hematopoiesis and neurological disorders [see Additional file 1, data sheet E]. In contrast, all other clusters which included genes expressed at higher levels in the STAT5Δ750-induced tumors contained the "cancer" category which assembled genes into specific tumorigenic processes. In this category, genes mediating "invasion", such as Adm, Itg3 and Igf2, converged to cluster 2. In contrast, most genes involved in tumor growth, transformation and differentiation were expressed at lower levels and were therefore allocated to cluster 3.

### Transgenic STAT5 variants exert distinct effects on a limited set of specifically affected genes

**Figure 3:**
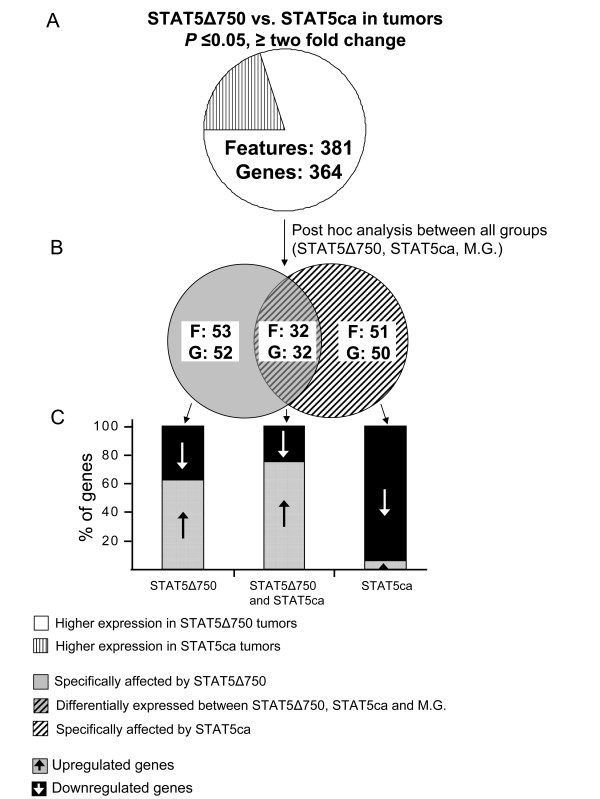
**Venn Diagrams of genes affected by the transgenic STAT5 variants relative to their expression in the mammary gland**. A. Levels of expression in the two sets of tumors were compared. B. Mammary gland expression levels were included in the analysis to identify genes that differ in their expression in a particular group (specifically affected genes). C. Up- or downregulation of gene expression in individual groups was defined.

Specifically affected genes differed in their expression between an individual tumor set and its counterpart, and between that set and the intact mammary gland. To identify genes which were specifically affected by each of the STAT5 variants, three mammary gland samples were added to the analysis, and the level of expression of the 364 genes exhibiting differential expression among STAT5-induced tumors was re-evaluated (Fig. 3). This analysis identified 52 genes associated with the effect of the STAT5Δ750 transgene [(STAT5Δ750 ≠ STAT5ca = mammary gland (M.G.)], 50 genes that were specifically affected by STAT5ca (STAT5ca ≠ STAT5Δ750 = M.G.), and 62 genes with differential expression in the three groups (STAT5Δ750 ≠ STAT5ca ≠ M.G., and [see Additional file 1, data sheet F]). Unexpectedly, 94% of the STAT5ca-affected genes (47 genes) were downregulated compared to their expression in the STAT5Δ750-induced tumors and the wild-type mammary gland. A considerable number of these genes could be associated with anti-proliferative or tumor-suppressive processes, including apoptosis inducers Edg3 and Jip-2 [37,38], differentiation inducers Msln, Il5ra and Ebf2 [39-41], Prp19 (also known as Pso4) which is involved in DNA repair [42], Ragef3 which blocks chemotaxis induced by angiogenic factors [43] and Tyrp, which is expressed in poorly metastatic breast cancer and whose downregulation augments metastasis [44].

A more equal deviation characterized the STAT5Δ750-affected genes. Only 38% of these genes were downregulated, whereas the rest were expressed at significantly higher levels compared to their expression in the STAT5ca-induced tumors or the wild-type mammary gland. In contrast to the STAT5ca-affected genes that were located in cluster 3, their STAT5Δ750 counterparts spread mainly among cluster 1 (20 genes, 38%) and cluster 2 (29 genes, 56%), with a residual presence in cluster 4 (3 genes, 6%). Upregulated and downregulated genes were annotated with different activities (Table 2). Upregulated genes affected angiogenesis, cell adhesion, cell-cell signaling, and progression through the cell cycle. Downregulated genes were involved in insulin receptor binding, DNA replication, chromatin remodeling and G-protein receptor pathways.

**Table 2:** Classification, annotation and expression levels of genes, specifically affected by the STAT5 variants.

**Specifically affected by BLG/STAT5ca**	
Ras small GTPase	Rasd1, Rasd2
GTPase activity	Rasd1 Rasd2 GNAO1
Rho GAPs	Chn1
G-protein	GNAO1
G-protein-coupled receptor	Edg3, **Adra2c**
Cytoskeleton protein	Tnni1, Cnn1, Rsnl2
GEFs	Rapgef3
Cytoskeleton rearrangement	Ptn, pip5k1b
Organization of microtubules	Rsnl2
Cell-cell recognition/signaling/adhesion	Msln, ptn, Rapgef3, **Adra2c**
Cell migration	Edg3
Mammary development	Ebf2, Gli2
Immune system/response	**Csf2ra**, il5ra, Foxji, H2-K1, EDG3
Neurons	Elavl3, Wnt8a, Gfra2, Phgdh
DNA damage response/cell cycle	Prp19 (DDR), Magea2 (inhibits p53 activation)
Apoptosis inhibition	Cflar
Tight junction	Mpdz, Gja7
Potassium channel	Kcne4
JNK signaling	JIP-2, Rapgef3, Mapk8
Transcription factor	Max
Defense response	Klra5, Klra22
Ribosome	Rps15a
Urea cycle	Otc
Signal transduction	Mapk1, JIP-2, Rapgef3, Mapk8, Gfra2, Adra2c, Rasd1, Rasd2, GNAO1, Chn1, EDG3, Wnt8a, JIP-2
Protein phosphates inhibitor activity	PPP1R2P9
Regulation of nucleotide and nucleic acid metabolism	Ddx3y
Bacterial cell wall degradation	lysmd2
Regulating hemoglobin oxygen affinity	Bpgm
Expressed in the early embryo	**Pramel6**
Not associated	Tyrp1, 1700065I17Rik, Serpinb6c, 2010003K11Rik, 4931412G03Rik (Trpd52l3)

**Specifically affected by BLG/STAT5Δ750**	

**Adm**, **Mrpplf4**, **Cyr61**	Angiogenesis
**Cspg2, Itgβ1, Itgβ3, Scgb1a1, Pcdhb5, Rin1, Lana5, Cyr61, Cav2**	Cell adhesion
**Itgβ1, Foxk1, Rnf134, Peg3**, Mkrn3	Progression through cell cycle
**Peg3, Kif5c, Pcdhb5, Ivns1abp**, Mkrn3	Neuron
**Foxk1, Rnf134, Peg3**	Transcription factor activity
**Gdi2, Rin1**	GTPase activator activity
Tec, Trhr, Oprl, V1ra9, 5930418K15Rik	G-protein-coupled receptor protein signaling pathway
**Adm, Wnt7b, Pcdhb5**	Cell-cell signaling
Irs4	Insulin receptor binding
Orc3l	DNA replication
**Eif2s2**	Translation initiation factor activity
**Cugbp1**	Translation repressor activity
**Cdv3, Cyr61**	Cell proliferation
Hsd17b1	Estradiol 17-beta-dehydrogenase activity
H2-Eb1, Tec, **Igbp1b**, **Nudcd1**	Immune response
**Nudcd1**, Mkrn3, Msmb, **Cav2**, **Gnmt**, Cryl1	Cancer-related
**Kif5c**	Microtubule-based movement
Abhd1	Hydrolase activity
Klf1	Chromatin remodeling
**Peg3**, Mkrn3	Apoptosis
**B3galt1**	Transferase activity, transferring glycosyl groups
**Igbp1b**	B-cell activation
**Krt2-17**, **Kif5c**	Cytoskeleton organization and biogenesis
**Pdss1**	Isoprenoid biosynthetic process
**Pi4k2b**	Phosphotransferase activity, alcohol group as acceptor
**Nol5**	Ribosome biogenesis and assembly
**Fabp5**	PPAR signaling pathway/lipid metabolic process
**Gnmt**	Breaks down the protein building block (amino acid) methionine
4930422J18Rik(SSB-1)	Ubiquitin cycle
**C80171**, 1810030O07Rik, Es2el, Pter	Not associated

In black–genes with higher expression in tumors induced by STAT5Δ750. In gray–genes with higher expression in tumors induced by STAT5ca.

### The expression of STAT5ca in individual tumors does not correlate with the expression of its specifically affected genes

Does the deviation in gene expression between the two sets of tumors result from an early transgenic STAT5 effect, or does continuously deregulated STAT5 expression in the tumors govern the gene-expression profile?

STAT5ca and STATΔ750 expression levels were analyzed by real-time PCR in the respective tumors. In contrast to measurable STAT5ca transcripts, STAT5Δ750 mRNA did not reach detection levels. There was no significant correlation between expression of STAT5ca in the tumors and that of any of its affected genes [see additional file 2, bottom row of upper section]. Interestingly, within the genes affected specifically by STAT5ca, a few groups were identified which were linked by significant correlations between the expressions of their individual members. The genes with the highest number of internal correlations are presented [see Additional file 2]. The largest such group included Mapk8 (top row), Wnt8b, Ptn, Pramel6, Klra5, Edg3, Ebf2 and Tyrp1. Significant gene-expression correlations were also detected among the STAT5Δ750-affected tumors, the largest group including Wnt7b, Scgb1a1, Itga3, Mrpplf4 and Cspg2. Analyses of these gene groups by DAVID [45] and IPA software could not assemble the individual members into a single metabolic pathway or identify a mutual effector.

Distinct distribution into clusters was observed when the specifically affected genes were allocated. With the list of highly correlated genes as a core, the STAT5ca-affected genes were assembled into a small region in the upper section of cluster 3 (Fig. 2), confirming a lower level of expression compared to both the STAT5Δ750-induced tumors and the wild-type mammary gland. In contrast, genes affected by STAT5Δ750 were distinctly located in clusters 2 and 1. Their location in cluster 2 implied a higher level of expression compared to those specifically affected by the STAT5ca transgene or the wild-type mammary gland. Their presence in cluster 1 as well reflected the more even distribution between upregulated and downregulated genes.

### Reproducing transgenic STAT5 effects in a "test set" of tumors

Our next step was to confirm the "transgenic signature" of the STAT5 variants in independent groups of tumors. From the list of 50 genes specifically affected by STAT5ca, six candidates were selected and their expression was analyzed by real-time-PCR in a separate set of tumors caused by the two STAT5 variants. This "test set" included seven tumors that had developed in BLG/STAT5ca-transgenic mice (four papillary adenocarcinomas, one squamous carcinoma and two poorly differentiated carcinomas) and six tumors that had developed in the BLG/STAT5Δ750 mice (one papillary adenocarcinoma, three micropapillary adenocarcinomas, one squamous carcinoma and one adenosquamous carcinoma). As demonstrated in Table 3, the differences between the expressions of genes affected by STAT5Δ750 vs. STAT5ca detected in the original set of tumors that was tested by the array analysis were comparable to those found in the "test set" which was subjected to real-time PCR.

**Table 3:** Validation of the differences in gene expression determined by the array analysis using real-time PCR of gene expression in a test set of tumors.

**Gene**	**Tumor set 1** **(Microarray analysis, arbitrary units)**			**Tumor set 2 (test set)** **(Real-Time PCR analysis, 2^-Δct ^× 1,000)**		
	BLG/STAT5Δ750 tumors	BLG/STAT5ca tumors	Fold change (BLG/STAT5Δ750/BLG/STAT5ca)	BLG/STAT5Δ750 tumors	BLG/STAT5ca tumors	Fold change (BLG/STAT5Δ750/BLG/STAT5ca)

Edg3	1.59 ± 0.21	0.43 ± 0.15	3.64	0.10 ± 0.02	0.04 ± 0.01	2.08
Mapk8	1.30 ± 0.19	0.51 ± 0.12	2.54	0.64 ± 0.189	0.43 ± .05	1.49
Wnt8a	1.41 ± 0.31	0.44 ± 0.14	3.15	1.41 ± 0.380	0.37 ± 0.21	3.75
Ptn	1.69 ± 0.48	0.46 ± 0.25	3.68	1.01 ± 0.219	0.48 ± 0.07	2.09
Foxk1	3.49 ± 0.97	0.86 ± 0.19	4.04	0.35 ± 0.118	0.25 ± 0.02	1.42
Tyrp1	1.37 ± 0.21	0.41 ± 0.17	3.33	2.22 ± 1.159	0.47 ± 0.18	4.71

## Discussion

The constitutively active Stat5 and its C-terminally truncated variant have been implicated in cancer in laboratory animals and humans. Here we established a model system, based on mice expressing the individual transgenic STAT5 variants, to study the distinct variants' oncogenic role in the mammary gland.

Stat5 mediates proliferation in the mammary gland of pregnant females [46], and different effects of its two variants have been demonstrated in transgenic mice during the reproductive cycle: STAT5ca-induced proliferation during pregnancy and delayed apoptosis and tissue remodeling during involution [11]. The opposite has been shown for the STAT5Δ750 variant [11,12]. This diversity results from the structural differences between the STAT5 variants which involves either forced activation or lack of TAD-mediated interactions with the proteins, such as CBP/p300, that recruit acetylases [47]. Both STAT5 variants seem to have a persistent effect, probably on chromatin structure and accessibility [16,48,49], which results in different profiles of gene expression in the developing tumors.

Differential expression of 364 genes was found between mammary tumors developed in BLG/STAT5ca- and BLG/STAT5Δ750-transgenic mice. These genes were involved in a set of cellular activities, many of which could still be associated with the two main processes mediated by Stat5 in the intact tissue: cell proliferation and cell death. The individual genes that established the specific mark of the STAT5 variants in the tumors were identified by gene-array analysis and their differential expression in the population was confirmed in a distinct "test set" of tumors using a different detection method, real-time PCR. Of special interest was the gene encoding the proto-oncogene Met, which was expressed in the STAT5Δ750-induced tumors at a 22-fold higher level than in their STAT5ca-induced counterparts. The product of Met is the hepatocyte growth factor receptor which encodes tyrosine-kinase activity. The ligand-activated cytoplasmic domain of the c-Met receptor induces growth motility, morphogenesis and angiogenesis [50]. In breast cancer, c-Met overexpression is associated with tumor progression (reviewed in [51]) and has an independent predictive value for poor survival, even in early-stage patients with negative lymph nodes [52]. Expression levels of Met were upregulated relative to intact tissue in the STAT5Δ750-induced tumors and downregulated in tumors developed in mice carrying the BLG/STAT5ca transgene. This differential expression suggests a more aggressive downstream cascade in the former. Other genes with exceptionally high expression levels in the STAT5Δ750-induced tumors, which were not affected by the STAT5ca variant, were proliferin and Igf2. Proliferin is a member of the prolactin family which is involved in progenitor cell expansion along the luminal and myoepithelial lineage [53] and Igf2 plays a pivotal role in fetal and cancer development by signaling via the IGF-I and insulin receptors, and activating the estrogen-signaling cascade [54]. Unfortunately, studies on these genes [55-57] do not include or base additional lists of genes with altered expression profiles that might be compared to our data and aid in delineating the pathway(s) involved in the tumorigenic effect of STAT5Δ750.

A group of genes regulating carbohydrate metabolism and transport could also be discerned. These genes were not associated with Stat5 effects in the intact mammary gland but were differentially expressed in the two sets of tumors. Their role in the tumors could be related to altered levels of metabolism (i.e. higher metabolic rate in the STAT5Δ750-induced tumors) rather than cancer growth *per se*.

Expression of an additional set of 14 genes linked the effects of the BLG/STAT5Δ750 and the BLG/STAT5ca transgenes to the resulting phenotypes of poorly differentiated carcinoma or highly differentiated papillary adenocarcinoma, respectively (r = 0.97) [see Additional file 1, data sheet G and [16]]. These genes cover a wide variety of cellular functions: calcium sensitivity of the myofibrils (troponin I and tropomyosin 1), interaction between the cell and the extracellular matrix (endomucin), normal adipose tissue development (lipin 3), lipid metabolism, cellular growth and apoptosis (caveolin 2), mRNA metabolism (5'-3' exoribonuclease 1), mitochondrial fatty acid oxidation (acyl coenzyme A dehydrogenase), as well as kinase and protease activities (TAU tubulin kinase 1 and corin, respectively). Taken together, their diverse expression is most likely involved in determining tumor phenotype, i.e. associating higher proportions of the poorly differentiated carcinomas or the highly differentiated papillary adenocarcinomas with the expression of STAT5Δ750 or STAT5ca, respectively [15].

Overall, 94% of the genes specifically affected by STAT5ca were downregulated relative to their expression in the host tissue. This contrasts with the more equal specific effect of the STAT5Δ750 variant and provides additional evidence for the distinct routes via which the two STAT5 variants impose their mark on tumor growth and maintenance. The high number of tumor and growth suppressors defined among the STAT5ca-downregulated genes vs. the potent oncogenes (Met, Igf2) that were induced by the STAT5Δ750 variant may serve to further distinguish the routes via which they initiate and maintain tumorigenesis.

Substantial downregulation of gene expression has been demonstrated in breast cancers with bone marrow (BM) micrometastasis [58], and during the molecular transition from organ-confined to metastatic prostate cancer [59]. Apparently, transcription repression is important for the metastatic process in these tissues. When compared with the list of genes downregulated by STAT5ca-, no overlap was observed for those associated with BM micrometastasis, and only four genes (Ptn, Cflar, Cnn1 and Mpdz) shared the list of downregulated genes mediating prostate metastasis. Thus, the resultant tumor characteristics are probably determined by a combination of the phenomenon of gene downregulation *per se *and the properties of the specifically affected genes (in this case by STAT5ca).

Our attempt to characterize the different roles of the STAT5 variants in mammary cancer development did not produce any evidence for the effect of transgenic STAT5 expression in the tumors. Neither distinct metabolic pathways nor a central mediator(s) were located. This suggests domination of an earlier STAT5 effect on the resulting gene-expression profiles. Several groups of genes, each composed of three to eight members, with significant internal correlations were identified among the sets specifically affected by STAT5ca and STAT5Δ750. This correlation was not associated with physical linkage. Within a single group, genes with correlated expression might control a distinct range of cellular functions such as angiogenesis (Ptn and Tyrp1 [60,61]), apoptosis (Cflar, [62]) and morphogenesis (Ebf2, [63]). Their comparable levels of expression, defined by their location in a confined region within the cluster, suggest that although these genes are mapped to several loci, they may colocalize to a shared transcription site. The concept of several loci being targeted to a shared transcription site where they generate "transcription factories" with similar levels of expression was proposed a few years ago and is reviewed in [64,65]. The relevance of this biological system to the specific STAT5 effects presented in this study remains to be determined. Regardless of the detailed mechanism involved in the effect of STAT5Δ750 and STAT5ca on tumor growth and maintenance, this study establishes the feasibility of identifying and distinguishing mammary tumors according the variants' signature on specific gene-expression profiles. To the best of our knowledge, this signature is specific to the effect of the STAT5 variants. However, its uniqueness will only be confirmed by the contribution of further gene-expression profiles that are specific for the effects of other oncogenes.

## Conclusion

The C-terminally truncated form of Stat5 and its constitutively active variant retain oncogenic potency that is conveyed via distinct pathways and probably initiated during early stages of tumor development. The detailed results of this study may have clinical implications regarding the decision of whether, and when, to use putative anti-Stat5 therapy [66].

## Abbreviations

BLG: β-lactoglobulin; Stat5: signal transducer and activator of transcription 5; STAT5: transgenic Stat5; STAT5ca: constitutively active STAT5; STAT5Δ750: truncated STAT5; TAD: transactivation domain.

## Competing interests

The authors declare that they have no competing interests.

## Authors' contributions

TE designed and performed the research and also helped in writing the manuscript. IB designed the research and wrote the manuscript.

## Supplementary Material

Additional file 1 **Supporting information. Primers list and bioinformatical analyses of differentially expressed genes in tumors caused by the STAT5ca and STAT5Δ750 variants**. Data sheet A. List of primers used to amplify coding regions of the following listed genes. Data sheet B. List of features with a significant (*P < 0.05*), over twofold difference in expression between STAT5Δ750- and STAT5ca-induced tumors. Data sheet C. IPA classification of the differentially expressed genes in tumors induced by STAT5ca and the STAT5Δ750 transgenes. List of genes comprising the process annotation to biological function and/or disease is presented. Data sheet D. Clustering of genes with a significant (*P < 0.05*), over twofold difference in expression in tumors caused by the two STAT5 variants. Data sheet E. Clustering and IPA classification of the differentially expressed genes in tumors caused by STAT5ca and STAT5Δ750 variants. Data sheet F. List of specifically affected genes and those differentially expressed in each of the tumor sets relative to the mammary gland. Data sheet G. Set of 14 genes with expression levels that associate the effect of STAT5Δ750 with the resulting carcinoma phenotype and the effect of STAT5ca with the papillary adenocarcinoma phenotype. Click here for file

Additional file 2 **Correlations between the expression of STAT5ca and its specifically affected genes, and among genes specifically affected by the STAT5 variants**. The table presents the correlation values between the expression levels of genes specifically affected by the STAT5ca and STAT5Δ750 variants in the tumors, and between the expression of STAT5ca and these genes. Click here for file
