# Supplementary material for: Distinct gene-expression profiles characterize mammary tumors developed in transgenic mice expressing constitutively active and C-terminally truncated variants of STAT5
Source: BMC Genomics. 2009 May 18;10:231. doi: 10.1186/1471-2164-10-231 (PMC2689279; doi:10.1186/1471-2164-10-231)
Supplement: Additional file 2 — Correlations between the expression of STAT5ca and its specifically affected genes, and among genes specifically affected by the STAT5 variants. The table presents the correlation values between the expression levels of genes specifically affected by the STAT5ca and STAT5Δ750 variants in the tumors, and between the expression of STAT5ca and these genes. [file 1471-2164-10-231-S2.doc]

**Additional file 2. Correlations between the expression of STAT5ca and its specifically affected genes, and among genes specifically affected by the STAT5 variants**

**Correlations between specifically affected genes in BLG/STAT5ca tumors**

| **Gene name** | **Affy. I.D.** | **Mapk8** | **Wnt8a** | **Max** | **Tyrp1** | **2210402C18Rik** | **Ptn** | **Ptn** | **Gja7** | **Pramel6** | **Prp19** | **Mapk1** | **Klra5** | **Cflar** | **Msln** | **Edg3** | **4930403L05Rik** | **Ebf2** | **STAT5ca** |
| --- | --- | --- | --- | --- | --- | --- | --- | --- | --- | --- | --- | --- | --- | --- | --- | --- | --- | --- | --- |
| **Mapk8** | **1420931_at** | 1 | 0.96*** | 0.68 | 0.72+ | 0 | 0.85* | 0.85* | -0.1 | 0.90** | 0.27 | -0.3 | 0.91** | 0.61 | -0.39 | 0.83* | -0.12 | 0.83* | 0.49 |
| **Wnt8a** | **1422228_at** | 0.96*** | 1 | 0.70+ | 0.66 | -0.1 | 0.79* | 0.74+ | -0.12 | 0.88** | 0.28 | -0.37 | 0.89** | 0.49 | -0.37 | 0.79* | -0.15 | 0.76* | 0.54 |
| **Max** | **1423501_at** | 0.68 | 0.70+ | 1 | 0.77* | 0.5 | 0.7 | 0.64 | -0.71 | 0.42 | 0.86* | -0.75 | 0.48 | 0.64 | -0.88 | 0.65 | -0.61 | 0.73 | 0.16 |
| **Tyrp1** | **1415861_at** | 0.72+ | 0.66 | 0.77* | 1 | 0.29 | 0.89** | 0.90** | -0.38 | 0.42 | 0.52 | -0.3 | 0.55 | 0.93** | -0.6 | 0.5 | -0.3 | 0.97*** | -0.06 |
| **2210402C18Rik** | **1428626_at** | 0 | -0.1 | 0.5 | 0.29 | 1 | 0.17 | 0.19 | -0.81 | -0.13 | 0.79* | -0.69 | -0.27 | 0.31 | -0.8 | 0.17 | -0.76 | 0.2 | -0.17 |
| **Ptn** | **1448254_at** | 0.85* | 0.79* | 0.7 | 0.89** | 0.17 | 1 | 0.96*** | -0.33 | 0.69 | 0.34 | -0.33 | 0.72+ | 0.73+ | -0.52 | 0.69 | -0.36 | 0.93** | 0.08 |
| **Ptn** | **1416211_a_at** | 0.85* | 0.74+ | 0.64 | 0.90** | 0.19 | 0.96*** | 1 | -0.23 | 0.66 | 0.29 | -0.19 | 0.75* | 0.84* | -0.48 | 0.71 | -0.2 | 0.94*** | 0.04 |
| **Gja7** | **1449094_at** | -0.1 | -0.12 | -0.71 | -0.38 | -0.82 | -0.33 | -0.23 | 1 | 0.07 | -0.87 | 0.85 | 0.12 | -0.25 | 0.92** | -0.31 | 0.92** | -0.26 | 0.27 |
| **Pramel6** | **1456269_at** | 0.90** | 0.88** | 0.42 | 0.42 | -0.13 | 0.69 | 0.66 | 0.07 | 1 | 0.03 | -0.26 | 0.83* | 0.25 | -0.16 | 0.75 | -0.08 | 0.6 | 0.69 |
| **Prp19** | **1460633_at** | 0.27 | 0.28 | 0.86* | 0.52 | 0.79* | 0.34 | 0.29 | -0.87 | 0.03 | 1 | -0.86 | -0.01 | 0.45 | -0.92 | 0.31 | -0.76 | 0.41 | 0.02 |
| **Mapk1** | **1419568_at** | -0.3 | -0.37 | -0.75 | -0.3 | -0.69 | -0.33 | -0.19 | 0.84* | -0.26 | -0.86 | 1 | 0 | -0.1 | 0.81* | -0.35 | 0.90** | -0.28 | -0.28 |
| **Klra5** | **1426140_x_at** | 0.91** | 0.89** | 0.48 | 0.55 | -0.27 | 0.72+ | 0.75* | 0.12 | 0.83* | -0.01 | 0 | 1 | 0.48 | -0.18 | 0.86* | 0.16 | 0.66 | 0.35 |
| **Cflar** | **1425687_at** | 0.61 | 0.49 | 0.64 | 0.93** | 0.31 | 0.73+ | 0.84* | -0.25 | 0.25 | 0.45 | -0.1 | 0.48 | 1 | -0.5 | 0.41 | -0.09 | 0.884** | -0.15 |
| **Msln** | **1460238_at** | -0.39 | -0.37 | -0.88 | -0.6 | -0.8 | -0.52 | -0.48 | 0.92** | -0.16 | -0.92 | 0.81* | -0.18 | -0.5 | 1 | -0.56 | 0.80* | -0.5 | 0.12 |
| **Edg3** | **1460661_at** | 0.83* | 0.79* | 0.65 | 0.5 | 0.17 | 0.69 | 0.71+ | -0.31 | 0.75+ | 0.31 | -0.35 | 0.86* | 0.41 | -0.56 | 1 | -0.24 | 0.56 | 0.24 |
| **4930403L05Rik** | **1419541_at** | -0.12 | -0.15 | -0.61 | -0.3 | -0.76 | -0.36 | -0.2 | 0.92** | -0.08 | -0.76 | 0.90** | 0.16 | -0.09 | 0.80* | -0.24 | 1 | -0.24 | 0.07 |
| **Ebf2** | **1449102_at** | 0.83* | 0.76* | 0.72+ | 0.97*** | 0.2 | 0.93** | 0.94*** | -0.26 | 0.6 | 0.41 | -0.28 | 0.66 | 0.88** | -0.5 | 0.56 | -0.24 | 1 | 0.13 |
| **STAT5ca** | **Stat5ca** | 0.49 | 0.54 | 0.16 | -0.06 | -0.17 | 0.08 | 0.04 | 0.27 | 0.69 | 0.02 | -0.28 | 0.35 | -0.15 | 0.12 | 0.24 | 0.07 | 0.13 | 1 |

**Correlations between specifically affected genes in BLG/STAT5Δ750 tumors**

| **Gene name** | **Affy. I.D.** | **Peg3** | **Abhd1** | **Wnt7b** | **Wnt7b** | **Msmb** | **4921532K09Rik** | **Hsd17b1** | **4930422J18Rik** | **Cdv3** | **Scgb1a1** | **Tec** | **Itga3** | **Mrpplf4** | **Krt2-17** | **Pter** | **Foxk1** | **Cspg2** |
| --- | --- | --- | --- | --- | --- | --- | --- | --- | --- | --- | --- | --- | --- | --- | --- | --- | --- | --- |
| **Peg3** | **1417356_at** | 1 | -0.26 | 0.16 | -0.18 | 0.98 | 0.93** | -0.08 | -0.71 | 0.94** | -0.23 | -0.17 | -0.11 | -0.17 | -0.86 | -0.57 | 0.23 | 0.2 |
| **Abhd1** | **1418148_at** | -0.26 | 1 | -0.38 | -0.45 | -0.13 | -0.27 | 0.92** | 0.03 | -0.32 | -0.34 | 0.41 | -0.63 | -0.39 | 0.43 | -0.14 | -0.82 | -0.44 |
| **Wnt7b** | **1420891_at** | 0.16 | -0.38 | 1 | 0.86* | 0.06 | 0.23 | -0.11 | -0.29 | 0.33 | 0.65 | -0.98 | 0.765+ | 0.67 | -0.24 | -0.09 | 0.07 | 0.99*** |
| **Wnt7b** | **1420892_at** | -0.18 | -0.45 | 0.86* | 1 | -0.27 | -0.13 | -0.35 | -0.1 | 0.07 | 0.92** | -0.84 | 0.93** | 0.92** | -0.07 | -0.04 | 0.3 | 0.83* |
| **Msmb** | **1421687_at** | 0.97*** | -0.13 | 0.06 | -0.27 | 1 | 0.84* | 0.02 | -0.79 | 0.88* | -0.28 | -0.04 | -0.25 | -0.23 | -0.87 | -0.67 | 0.19 | 0.09 |
| **4921532K09Rik** | **1429655_at** | 0.93** | -0.27 | 0.23 | -0.13 | 0.84* | 1 | -0.05 | -0.45 | 0.92** | -0.28 | -0.3 | 0 | -0.22 | -0.66 | -0.31 | 0.07 | 0.26 |
| **Hsd17b1** | **1449392_at** | -0.08 | 0.92** | -0.11 | -0.35 | 0.02 | -0.05 | 1 | -0.06 | -0.15 | -0.38 | 0.14 | -0.56 | -0.42 | 0.33 | -0.11 | -0.92 | -0.16 |
| **4930422J18Rik** | **1449752_at** | -0.71 | 0.03 | -0.29 | -0.1 | -0.791+ | -0.45 | -0.06 | 1 | -0.67 | -0.2 | 0.19 | 0.05 | -0.24 | 0.87* | 0.92** | -0.27 | -0.3 |
| **Cdv3** | **1451100_a_at** | 0.94** | -0.32 | 0.33 | 0.07 | 0.88* | 0.92** | -0.15 | -0.67 | 1 | 0.02 | -0.37 | 0.17 | 0.08 | -0.82 | -0.58 | 0.26 | 0.35 |
| **Scgb1a1** | **1452543_a_at** | -0.23 | -0.34 | 0.65 | 0.92** | -0.28 | -0.28 | -0.38 | -0.2 | 0.02 | 1 | -0.61 | 0.84* | 0.99*** | -0.12 | -0.26 | 0.41 | 0.62 |
| **Tec** | **1460204_at** | -0.17 | 0.41 | -0.98 | -0.85 | -0.04 | -0.3 | 0.14 | 0.19 | -0.37 | -0.61 | 1 | -0.8 | -0.63 | 0.19 | 0 | -0.05 | -0.97 |
| **Itga3** | **1460305_at** | -0.11 | -0.63 | 0.76+ | 0.93** | -0.25 | 0 | -0.56 | 0.05 | 0.17 | 0.84* | -0.8 | 1 | 0.86* | -0.07 | 0.09 | 0.43 | 0.75+ |
| **Mrpplf4** | **1427760_s_at** | -0.17 | -0.39 | 0.67 | 0.92** | -0.23 | -0.22 | -0.42 | -0.24 | 0.08 | 0.99*** | -0.63 | 0.86* | 1 | -0.18 | -0.28 | 0.46 | 0.64 |
| **Krt2-17** | **1427154_at** | -0.86 | 0.43 | -0.24 | -0.07 | -0.87 | -0.66 | 0.33 | 0.87* | -0.82 | -0.12 | 0.19 | -0.07 | -0.18 | 1 | 0.76+ | -0.58 | -0.28 |
| **Pter** | **1453578_at** | -0.57 | -0.14 | -0.09 | -0.04 | -0.67 | -0.31 | -0.11 | 0.92** | -0.58 | -0.26 | 0 | 0.09 | -0.28 | 0.76+ | 1 | -0.24 | -0.07 |
| **Foxk1** | **1421759_a_at** | 0.23 | -0.82 | 0.07 | 0.3 | 0.19 | 0.07 | -0.92 | -0.27 | 0.26 | 0.41 | -0.05 | 0.43 | 0.46 | -0.58 | -0.24 | 1 | 0.12 |
| **Cspg2** | **1427257_at** | 0.2 | -0.44 | 0.99*** | 0.83* | 0.09 | 0.26 | -0.16 | -0.3 | 0.35 | 0.62 | -0.97 | 0.75+ | 0.64 | -0.28 | -0.07 | 0.12 | 1 |

+ p ≤ 0.08; * p ≤ 0.05; **p ≤ 0.01; *** p ≤ 0.001
